# Aqueous extract of *Piper sarmentosum *decreases atherosclerotic lesions in high cholesterolemic experimental rabbits

**DOI:** 10.1186/1476-511X-9-44

**Published:** 2010-04-30

**Authors:** Adel A Amran, Zaiton Zakaria, Faizah Othman, Srijit Das, Santhana Raj, Nor-Anita MM Nordin

**Affiliations:** 1 Department of Physiology, Faculty of Medicine, Universiti Kebangsaan Malaysia, Jalan Raja Muda Abdul Aziz, Kuala Lumpur 50300, Malaysia; 2 Department of Anatomy, Faculty of Medicine, Universiti Kebangsaan Malaysia, Jalan Raja Muda Abdul Aziz, Kuala Lumpur 50300, Malaysia; 3 Unit Electron Microscopy, Institute of Medical Research, Jalan Pahang, 50588 Kuala Lumpur, Malaysia

## Abstract

**Background:**

*Piper sarmentosum *(P.s) has flavonoid component in its leaves which has antioxidative effect. To date, its effect on atherosclerosis has not been studied histologically.

**Aim:**

The study aimed to investigate the effect of *P.s *on atherosclerotic changes in hypercholesterolemic rabbits.

**Methods:**

Forty two male New Zealand white rabbits were divided into seven groups. C - control group fed normal rabbit chow, CH - cholesterol diet (1% cholesterol), W1 - 1% cholesterol with water extract of *P.s *(62.5 mg/kg), W2 - 1% cholesterol with water extract of *P.s *(125 mg/kg), W3 - 1% cholesterol with water extract of *P.s *(250 mg/kg), W4 - 1% cholesterol with water extract of *P.s *(500 mg/kg) and Smv - 1% cholesterol supplemented with simvistatin drug (1.2 mg/kg). All rabbits were treated for 10 weeks. Following 10 weeks of supplementation, the animals were sacrificed and the aortic tissue was taken for histological study.

**Results:**

Rabbits fed only with high cholesterol diet 1% cholesterol (CH) showed focal fatty streak lesions compared to the C group and 1% cholesterol supplemented with simvistatin drug (Smv) group. Atherosclerotic lesions in the 1% cholesterol group supplemented with *P.s *(500 mg/kg) i.e. W4 group showed significant reduction (30 ± 6.0%, p < 0.05) in fatty streak compared to the high cholesterol group (85.6 ± 4.1%) under Sudan IV stain. The atherosclerotic lesions under transmission electron microscope showed reduction in foam cells in the treatment groups compared to the CH groups.

**Conclusion:**

Administration of *P.s *extract has protective effect against atheroscleros

## Introduction

*Piper sarmentosum *belongs to the family *Piperaceae *and it is widely cultivated in tropical and subtropical countries. The plant is popular due to its culinary and medicinal properties. In different parts of the world, P.s has been used traditionally to cure many diseases [1]. Phytochemically, the plant contains constituents likes alkaloids (amide, flavonoids, pyrones) [2] and it has also been reported to possess pharmacological properties like anti-tuberculosis [3] anti cancer [4], anti-angiogenic [5], hypoglycaemic [6], antimalarial [7], antioxidant [8], neuromuscular blocker [9] and antiamebic [10]. Due to these properties, the plant has a great potential of commercialization as medicinal plant in Malaysia and South-East Asia.

Today, herbal medicine has grown in popularity all over the world. Many individuals have resorted to herbal remedies in their daily life especially in developing countries, because of absence of adverse effects and cost effectiveness [11].

Atherosclerosis is the major cause of morbidity and mortality in the developing and developed countries [12]. The magnitude of this problem is profound as atherosclerosis claims more lives than all types of cancer combined and the economic costs are considerable. Atherosclerosis is characterized by the accumulation of cholesterol deposits in macrophages in large- and medium-sized arteries. This deposition leads to a proliferation of certain cell types within the arterial wall that gradually impinge on the vessel lumen and obstruct the blood flow. This process may proceed for decades until an atherosclerotic lesion is formed. As a result, the blood flow is disrupted and deep arterial wall components are exposed to flowing blood, leading to thrombosis and compromised oxygen supply to target organs such as the heart and brain. Various harmful agents such as smoking, hypertension, and diabetes may play an important role in initiating chronic inflammation, which predisposes vulnerable plaque to rupture and cause thrombosis. The endothelial dysfunction and inflammation causes not only the initial stage of the atherosclerotic process but also leads to atherosclerotic plaque development.

Atherosclerosis can be modified from chronic inflammation induced by lipids [13]. The arterial lesions in human atherosclerosis closely resembles that of the cholesterol fed rabbits. The endothelial dysfunction during atherosclerotic process was reported earlier by Ross (1999) [14]. Keeping in view the above facts, the present study was designed to observe the process of atherosclerosis in experimental rabbits and the protective role of P.s extract in arresting such atherosclerosis.

## Materials and methods

### Animals and experimental protocol

Prior ethical approval was obtained from the Animal Ethics Committee, Universiti Kebangsaan Malaysia. Forty two male New Zealand White rabbits with body weight of 1.8 ± 2 kg were obtained from East Asia Rabbit Corporation Sdn. Bhd. Malaysia, and were housed separately in cages in an air-conditioned room with a12-h light/dark cycle. All animals were fed with pellet for two weeks before starting the experiment, allowed drinking water *ad libitum *and fed vegetable diet comprising of cabbage and carrot once per week. The rabbits were then randomly divided into seven groups; control group (C; n = 6) rabbits was fed the standard diet, atherogenic rabbits group (CH; n = 6) was fed the standard diet enriched with 1% cholesterol, treatment groups (W1;n = 6, W2; n = 6, W3; n = 6 and W4; n = 6), were fed with standard diet enriched with 1% cholesterol plus different doses of water extract of P.S (62.5, 125, 250 and 500 mg/kg/day) respectively. The dosage pattern of 125, 250 and 500 mg/kg/day was adopted from an earlier protocol [15]. Admittedly, we did not perform any dose - response curve. The simvistatin group (Smv; n = 6) was fed with the standard diet enriched with 1% cholesterol plus simvistatin drug (1.2 mg/kg/day, Merck, NJ) [16]. The experiment was continued till 10 weeks. At the end of 10 weeks, the animals were fasted overnight and sacrificed by intravenous injection of pentobarbital (Nembutal, Abbott Laboratories, North Chicago, IL, 50 mg/kg body weight) and the aortic tissue was collected for histological studies.

### *P.s* extract preparation

The leaves of *P.s *were extracted by aqueous method by Furley Marketing Sdn,Bhd, Malaysia. The water extract sample was then sent to the laboratory of Faculty of Pharmacy, where the freeze dried powdered extract was prepared and the powder extract was stored in dark bottles and kept in 4°C until used. The powder was mixed with 5 ml of water to dissolve it and then administered to the rabbits.

### High cholesterol diet

Analytical pure cholesterol powder (Sigma Chemical Co., St. Louis, USA) was mixed with the rabbit chow pellet (1% cholesterol, w/w, in food pellet). For each 200 g of grounded rabbit chow pellet, 2 g of cholesterol was added and mixed with a 34 ml of chloroform where cholesterol was dissolved in 99.9% chloroform and then mixed with grounded rabbit chow pellet. Chloroform was evaporated by exposing the diets as a thin layer at 50°C in oven [17].

### Quantification of aortic atherosclerosis

After sacrificing the rabbits, the abdominal aorta was removed and then dissected longitudinally and the aorta was cut and stained with Sudan IV to evaluate the intimal lipid lesion, quantitatively. This quantitative measurement was performed by calculating the percentage of atherosclerotic lesions. The aortic tissue was fixed in 10% buffered formalin for one day, then the aorta was stained with Sudan IV for 15 minutes, followed by 2 minutes in 70% methanol alcohol and then washed with water for one hour. The result was measured as percentage of the lesion area using Video Test T-Morphology 5.1 software with camera (Pixlink).

Another part of the aorta was used for H&E staining. It was fixed in 10% phosphate buffered formalin and then embedded in paraffin. From each sample, serial sections were make (3-5 sections/aorta) by using microtome (Leica RM2135). The thickening of intima and media were measured and the ratio between tunica intima and tunica media were calculated. The 100-fold-magnification optical microscopic images were obtained using-Pixelink color camera (USA) with a computerized image analysis system Video Test T-Morphology 5.1 software with light microscope (Leica DM RXA2; German).

### Transmission electron microscopy

The aorta was fixed with 1% osmium tetroxide for 60 minutes, dehydrated in an ethanol series diluted and embedded in standard epoxy. After polymerization, the specimens were sectioned. The section was stained with lead citrate and uranyl acetate and examined under electron microscope (Tecnai G2, FEI Company) at an accelerating voltage of 100 kV.

### Statistical analysis

Statistical analysis was carried out using the SPSS statistical package version 12(SPSS Inc. USA). Normal distribution of all variables was examined by Kolmogrov-Smirnov test. The results showed that all variables were normally distributed. All data was analyzed using ANOVA test.

## Results

### Histological analysis with Sudan IV stain

**Figure 1:**
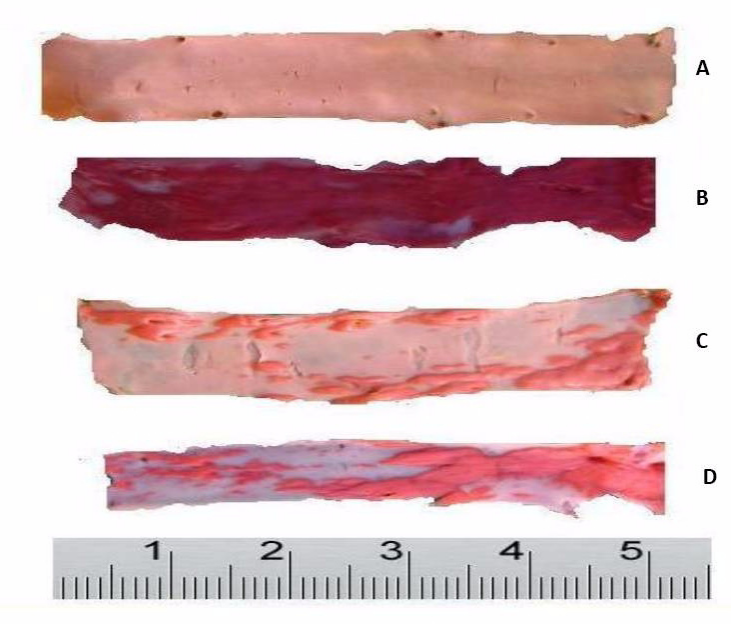
**Photograph of intimal surface of aorta by Sudan IV**. A) control group, there was no visible lesions (B) Atherogenic group, showed stainable lipid deposit covered most of the intimal surface, (C) W4 and Smv groups showed less lipid deposit (D). W1, W2 & W3 groups, showed there were more lesions as compared to W4; Note: marked red colour stain denotes lipid deposits.

In the C group which was devoid of cholesterol diet, no atherosclerotic lesion was observed. The atherosclerotic lesions in CH group was significantly increased (85.5 ± 4.0%) compared to the C group (p < 0.05) due to cumulative exposure of the aortic walls to cholesterol (Fig. 1). As a result of treatment with *P.s *in W1 group, the atherosclerotic lesion reduced (65.0 ± 7.0) but it was not significant compared to the CH group. The changes in the atherosclerotic lesion was significant in W4 group (29.0 ± 6.0, p ≤ 0.05), compared to the CH group. The treatment in Smv group also showed significant reduction in atherosclerotic lesion area (27.0 ± 2.6) (Table 1).

**Table 1:** Effect of P.s on the atherosclerotic lesion by Sudan IV and intimal ratio

Group	C	CH	W1	W2	W3	W4	smv
**Sudan IV %**	0 ± 0	85.5 ± 4.0	65.0 ± 7.0	85.0 ± 7.0	63.5 ± 12.0	29.0 ± 6.0*	27.0 ± 2.6*
**Intima ratio**	0 ± 0	1.7 ± 1.1	0.9 ± 0.6	1.5 ± 0.9	0.7 ± 0.3	0.4 ± 0.5*	0.1 ± 0.2*

All values mean ± SD

*P ≤ 0.05 by Kruskal-Wallis test as compared to CH group

### Histological analysis with Haematoxylin & Eosin stain

**Figure 2:**
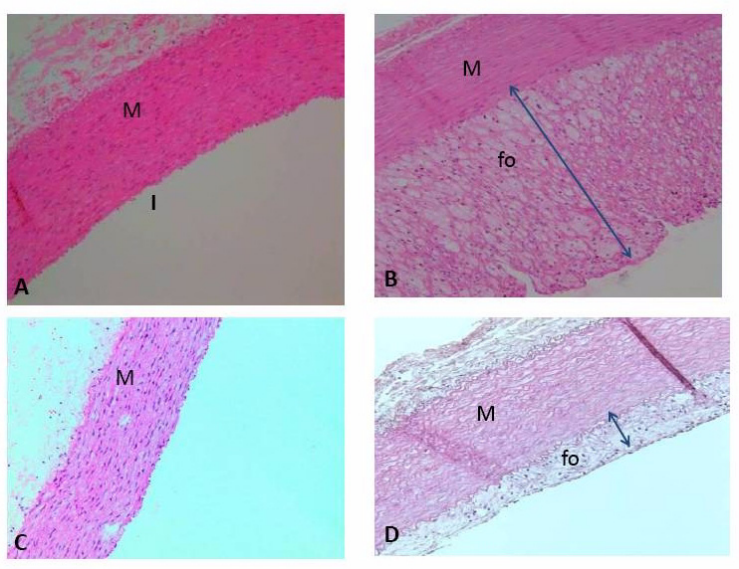
**Atherosclerotic morphology changes in rabbit abdominal aorta**. (A) C group (B) CH group the foam-cell reduced the lumen due to infiltration of the intima by foam cells (C) Smv group  (D) W4 group the foam cell layer and thickening of intima less reduction of the vascular lumen, IE; internal elastic lamina. Fo; foam cell, M; Media, I; intima. Orginal magnification ×100.

Histological examination showed thickening of tunica intima: tunica media of the abdominal aorta in the six groups of rabbits that received 1% cholesterol daily (Fig. 2). Three to four cross sections of the aorta were used from each rabbit and the average were taken. In CH group there was significant increased in the ratio (1.7 ± 1.1) compared to the C group. In group W1 & W2 there was less thickening of tunica intima compared to CH group but in the W3, W4 & Smv groups, there was significant reduction of the tunica intima layer. The ratio of tunica intima: tunica media also changed significantly (p < 0.05) (Table 1).

### Histological analysis under Transmission electron microscopy

**Figure 3:**
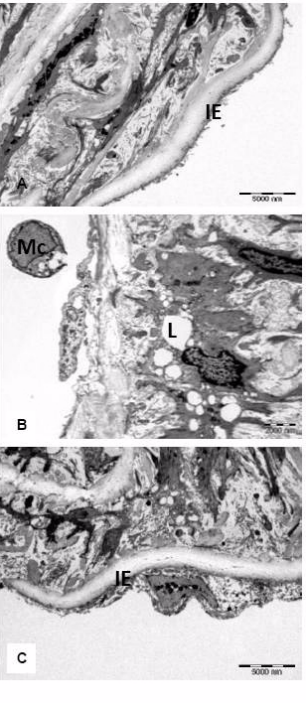
**Transmission electron photograph from a lesion in the luminal abdominal aorta**: (a) In control group there was no intimal thickening(IE) (Bar = 5000 nm), (B) CH group rabbit, there is marked irregularity of the surface caused by the presence of cells containing many (L) lipid filled vacuoles,(Mc) monoclonal cells were also seen on the endothelial surface, (Bar = 2000 nm) (c) treatment with *P.s,* there was less intimal thickening and only one cell containing lipid compared to CH group (Bar = 5000 nm).

In transmission electronic microscopy study, we selected blindly the sample from each group for histological analysis for control groups, no changes were observed in the intima surface layer. In CH group, there were foam cells in the intima layer. The tunica intima was thickened compared to other groups that led to reduction of vascular lumens. In treatment groups W3, W4 & Smv, there were less foam cells and no fat cells were observed. There was less tunica intima thickening (Fig. 3).

## Discussion

Hypercholesterolemia is a major risk factor for coronary heart disease, and the Framingham study had reported that a 1% increase of plasma cholesterol level is equivalent to a 2% elevation of coronary heart disease incidence [18]. The present study showed that dietary treatment of rabbits with high-cholesterol diets caused atherosclerotic lesions in an animal model and these findings were in accordance with earlier studies [19]. Feeding with excess amount of cholesterol causes rapid hyperlipidemia and atherosclerosis [20,21].

In the present study, we observed that the severity of the atherosclerosis lesions in aorta was associated with hypercholesterolemia which was in accordance with past investigations [22]. The results of the present study also showed that hypercholesterolemic diet produced tunica intimal thickening that contained foam cells which was even reported by past researchers [22]. Hypercholesterolemia is also one of the important factors that causes endothelial dysfunction in human arteries [23]. Atheromatous lesions develop in the subendothelial space due to the accumulation of cholesterol ester in forming foam cells. The mechanism of foam cell formation was unclear because macrophages have few LDL receptors but there is much evidence that oxidized LDL is responsible for cholesterol loading of macrophages foam cell formation and atherosclerosis.

*P.s *is a herb which is known to possess anti-inflammation and antioxidant properties. The active extract of *P. sarmentosum *contains natural antioxidants like Naringenin (75.7%), Hesperitin (91.7%), Taxifolin/Dihydroquercitin (90.9%) and Quercetin (98.1%) which have high superoxide scavenging action [8]. Histological results indicate that *P.s *significantly reduced atherosclerotic lesions in abdominal aorta of the *P.s *treated group compared to the high-cholesterol groups. Inflammation plays an important role in the development of atherosclerosis, hence, significant reduction in inflammatory lesion may be due to anti-inflammatory action of *P.s*.

The present study demonstrated that *P.s *possessed antiatherogenic activity. Supplementation of *P.s *reduces the thickening of the tunica intima layer and decreased the atherosclerotic lesion. The reduction in the tunica intima thickening due to supplementation with *P.s *was not clearly understood. The protective activity of *P.s *on atherosclerotic lesion may be attributed to its antioxidant action because it reduces the production of free radicals, decreasing the oxidized LDL and alleviating the subsequent damage to the heart tissue [24]. The effect may also be due to active compound like naringenin which has antiatherogenic effect [25].

The oxidative modification of LDL plays an important role in the development of atherosclerosis [26,27]. There are many antioxidant components in *P.s *like flavonoids which have potent action in protecting LDL from oxidation. The antioxidant activity of *P.s *extract was found to have scavenger free radical activity 70.6% [8]. Other studies have also shown that consumption of flavonoid antioxidant is inversely related to the risk of developing coronary heart disease [28]. Past research reports showed a link between flavonoid and atherosclerosis because of the antioxidant activities of phynolic compounds like flavonoid which inhibits the aggregation and adhesion of platelets in the blood [25]. It has been also shown that flavonoids reduce LDL oxidation, which is an important step in atherogenesis [29].

The present results in experiment rabbits, although not directly applicable to human, suggest that *P.s *may be effective as an anti-atherosclerotic agent. We observed foam cells under both light and transmission electron microscope in cholesterol group that caused reduction to the lumen surface. which was similar to results reported in past studies [30]. The protective effect of flavonoids against chronic diseases have been attributed to their free radical-scavenging property. Interestingly, in the case of CVD, flavonoids have been shown to reduce low density lipoprotein (LDL) oxidation which is an important step in the pathogenesis of atherogenesis [29].

## Conclusion

The results of the present study demonstrated that aqueous extract of *P.s *reduced atherosclerotic lesion in the aorta of hypercholesterolemic rabbit. Thus, *P.s *may be used effectively as an anti-cholesterolemic agent in the development of atheromatous lesions. Further studies may be needed to corroborate such facts.

## Abbreviations

(*P.s*): Piper sermentosum; (C): control group; (CH): Atherogenic group; (W1): 1% cholesterol together with water extracts of P.S with doses 62.5 mg/kg group; (W2): 1% cholesterol together with water extracts of P.S with doses 125 mg/kg; (W3): 1% cholesterol together with water extracts of P.S with doses 250 mg/kg; (W4): 1% cholesterol together with water extracts of P.S with doses 500 mg/kg; (Smv): 1% cholesterol supplemented with simvistatin drug 1.2 mg/kg; (HO-1): heme oxygenase-1; LDL: Low Density Lipoprotein; CVD: Cardiovascular Disease.

## Competing interests

The authors declare that they have no competing interests.

## Authors' contributions

ZZ was involved in supervising the project, and revising the manuscript critically for important intellectual content. AA carried out all aspects of experiments, design and data analysis, and drafted the manuscript and revising it critically for important intellectual content. FO was involved in interpreting the results and revising it critically for important intellectual content. SD was involved in histological interpretation of results, design, grammars, technical assistance in preparing the manuscript. SR was involved in the transmission electron microscope and interpretation of figures of TEM & NMN was involved in the extraction of *Piper sarmentosum* and revising the manuscript critically for important intellectual content. All authors have read and approved the final manuscript.

## References

[B1] Saralamp P Chuakul W Temsiririrkkul R Clayton T Medicinal plants in Thailand Bangkok, Amarin 1996 1 151

[B2] Tutiwachwuttikul P Phansa P Pootaeng-on Y Tylor WC Chemical constituents of the roots *Piper sarmentosum* Chem Pharm Bull 2006 54 suppl 2 149 151 10.1248/cpb.54.149 16462055

[B3] Hussain K Ismail Z Sadikun A Ibrahim P Analysis of proteins, polysaccharides, glycosaponins contants of *Piper sarmentosum *Roxb. and anti-TB evaluation for bio-enhancing/interation effects of leaf extracts with isonazid(INH) Natural Product Radiance 2008 7 Suppl 5 204 208

[B4] Shahrul HZA Wan Haifa HWO Zaidah ZA Muhd FS Sahidan S Rohaya MAW Intrinsic anticarcinogenic effects of Piper sarmentosum ethanolic extract on a human hepatoma cell line Cancer Cell Int 2009 10.1186/1475-2867-9-6 PMC2667431 19257877

[B5] Hussain K Ismail Z Sadikun A Ibrahim P Malik A in vitro antiagiogenesis activity of standrized extract of *Piper sarmentosum *Roxb J Ris Kim 2008 1 146 150

[B6] Penchom P Suwan ST Rungravi T Hirosh W Jeevan KP Shigetoshi K Hypoglycemic effect of the water extract of *Piper sarmentosum *in rats J Ethnopharmacol 1998 60 27 32 10.1016/S0378-8741(97)00127-X 9533429

[B7] Najib Nik A Rehman N Furuta T Kojima S Takane K Ali MM Antimalarial activity of extracts of Malaysian medicinal plants J Ethnopharmacol 1999 64 249 254 10.1016/S0378-8741(98)00135-4 10363840

[B8] Vimala S Mohd IA Abdull RA Rohana S Natural Antioxidants: *Piper sarmentosum *(Kadok) and *Morinda elliptica* Mal J Nutr 2003 9 suppl 1 41 51 22692531

[B9] Ridititid W Rattanaporm W Thaina P Chittrakaran S Sunbhanich M Neuromuscular blocking activity of methanolic extract of *Piper sarmentosum *leaves in the rat phrenic nerve hemi diaphragm preparation J Ethnopharmacol 1998 61 135 142 10.1016/S0378-8741(98)00025-7 9683344

[B10] Sawangjiaroen N Sawangjiaroen K Poonpanang P Effect of *Piper longum *fruit, *Piper sarmentosum *root and Quercus infectoria nut gall on caecal amoebiasis in mic J Ethnopharmacol 2004 91 357 360 10.1016/j.jep.2004.01.014 15120461

[B11] Ernst E Harmless Herbs? A review of the recent literature Am J of Med 1998 104 170 178 10.1016/S0002-9343(97)00397-5 9528737

[B12] Stocker R Keaney JF Role of oxidative modifications in atherosclerosis Physiol Rev 2004 84 1381 1478 10.1152/physrev.00047.2003 15383655

[B13] Glass CK Witztum JL Atherosclerosis: the road ahead Cell 2001 104 503 516 10.1016/S0092-8674(01)00238-0 11239408

[B14] Ross R Atherosclerosis: an inflammatory disease N Engl J Med 1999 340 115 126 10.1056/NEJM199901143400207 9887164

[B15] Sawangjaroen N Sawangjaroen K Poonpanang P Effects of *Piper longum *fruit, *Piper sarmentosum *root and *Quercus infectoria *nut gall on caecal amoebiasis in mice J Ethnopharmacol 2004 91 357 360 10.1016/j.jep.2004.01.014 15120461

[B16] Tsung ML Mei SL Tsai FC Nen CC Effect of simvastatin on left ventricular mass in hypercholestrolemic rabbits Am J Physiol Heart Circ Physiol 2005 288 1352 1358 10.1152/ajpheart.00527.2003 15486036

[B17] Julie HC Johnny LE Nicole JS Gordon RC Molecular Basis by which Garlic suppresses Atherosclerosis Journal of Nutrition 2001 131 1006 1009 10.1093/jn/131.3.1006S 11238806

[B18] Kannel WB Dawber TR Kagan A Revostski N Strokes J Factors of risk in the development of coronary heart disease-six year follow-up experience: the Framingham Study Ann Inter Med 1961 55 33 50 10.7326/0003-4819-55-1-33 13751193

[B19] Yi PS Nancy CL William WP Clarie BH Effect of cholesterol diet on vascular function and atherogenesis in rabbits Exp Biol Med 2000 224 166 171 10.1046/j.1525-1373.2000.22416.x 10865232

[B20] Amran AA Zaiton Z Faizah O Morat P Effects of *Garcinia atroviridis *on serum profiles and atherosclerotic lesions in the aorta of guinea pigs fed a high cholesterol diet Singapore Med J 2009 50 Suppl 3 295 299 19352574

[B21] Yamakoshi J Piskula MK Izumi T Tobe K Saito M Kataoka S Obata A Kikuchi M Isoflavone aglycone-rich extract without soy protein attenuates atherosclerosis development in cholesterol-fed rabbits J Nutr 2000 130 1887 1893 10917898 10.1093/jn/130.8.1887

[B22] Prasad K Kalra J Lee P Oxygen free radicals as a mechanism of hypercholesterolemic atherosclerosis: effects of probucol Int J Angiol 1994 3 100 112 10.1007/BF02014924

[B23] Minor RL Myers PR Guerra RJ Bates JN Harrison DG Diet-induced atherosclerosis increases the release of nitrogen oxides from rabbit aorta J Clin Invest 1990 86 2109 2116 10.1172/JCI114949 2254462 PMC329851

[B24] Zhang Z Chang Q Zhu M Huang Y Ho WKK Chen ZY Characterization of antioxidants present in hawthorn fruits J Nutr Biochem 2001 12 144 152 10.1016/S0955-2863(00)00137-6 11257463

[B25] Seong CC Hyo SK Tae SJ Song HB Young BP Naringin Has an Antiatherogenic Effect With the Inhibition of Intercellular Adhesion Molecule-1 in Hypercholesterolemic Rabbits J Cardiovasc Pharmacol 2001 38 947 955 10.1097/00005344-200112000-00017 11707699

[B26] Steinberg D Parthasarathy S Carew TW Knoo JC Witztum JL Beyond cholesterol: modification of low-density lipoprotein that increase its atherogenicity N Engl J Med 1989 320 915 924 2648148 10.1056/NEJM198904063201407

[B27] Jialal I Devaraj S Low-density lipoprotein oxidation, antioxidants, and atheroscelerosis: a clinical biochemistry perspective Clin Chem 1996 4 498 506 8605665

[B28] Hertog MGL Feskens EJM Hollman PCH Katan MB Kromhout D Dietary antioxidant flavonoids and risk of coronary heart disease: The Zutphen Elderly Study Lancet 1993 342 1007 1011 10.1016/0140-6736(93)92876-U 8105262

[B29] De Whalley CV Rankin SM Hoult JR Jessup WDS Flavonoids inhibit the antioxidative modification of low density lipoproteins Biochem Pharmacol 1990 39 1743 1749 10.1016/0006-2952(90)90120-A 2344371

[B30] Juan JS Ana IR Rosa H Blanca R Emilio R Teresa T Alberto T Jose MR Alterations in the choroid in hypercholesterolemic rabbits: reversibility after normalization of cholesterol levels Exp Eye Res 2007 84 suppl 3 412 22 17178413 10.1016/j.exer.2006.10.012

